# Selenium and Coenzyme Q10 Supplementation Improves Renal Function in Elderly Deficient in Selenium: Observational Results and Results from a Subgroup Analysis of a Prospective Randomised Double-Blind Placebo-Controlled Trial

**DOI:** 10.3390/nu12123780

**Published:** 2020-12-09

**Authors:** Urban Alehagen, Jan Aaseth, Jan Alexander, Kerstin Brismar, Anders Larsson

**Affiliations:** 1 Division of Cardiovascular Medicine, Department of Health, Medicine and Caring Sciences, Linköping University, SE-581 85 Linköping, Sweden; 2 Research Department, Innlandet Hospital Trust, N-2381 Brumunddal, Norway; jaol-aas@online.no; 3 Norwegian Institute of Public Health, N-0403 Oslo, Norway; Jan.Alexander@fhi.no; 4 Department of Molecular Medicine and Surgery, Karolinska University Hospital, Karolinska Institutet, SE-17176 Stockholm, Sweden; Kerstin.Brismar@ki.se; 5 Department of Medical Sciences, Uppsala University, SE-751 85 Uppsala, Sweden; anders.larsson@medsci.uu.se

**Keywords:** renal function, micronutrients, supplementation, elderly, cardiovascular mortality, selenium, coenzyme Q10

## Abstract

A low selenium intake is found in European countries, and is associated with increased cardiovascular mortality. There is an association between selenium level and the severity of kidney disease. An association between inflammation and selenium intake is also reported. The coenzyme Q10 level is decreased in kidney disease. The aim of this study was to examine a possible association between selenium and renal function in an elderly population low in selenium and coenzyme Q_10_, and the impact of intervention with selenium and coenzyme Q_10_ on the renal function. The association between selenium status and creatinine was studied in 589 elderly persons. In 215 of these (mean age 71 years) a randomised double-blind placebo-controlled prospective trial with selenium yeast (200 µg/day) and coenzyme Q_10_ (200 mg/day) (*n* = 117) or placebo (*n* = 98) was conducted. Renal function was determined using measures of glomerular function at the start and after 48 months. The follow-up time was 5.1 years. All individuals were low on selenium (mean 67 μg/L (SD 16.8)). The changes in renal function were evaluated by measurement of creatinine, cystatin-C, and the use of the Chronic Kidney Disease Epidemiology Collaboration (CKD-EPI) algorithm, and by the use of T-tests, repeated measures of variance and ANCOVA analyses. An association between low selenium status and impaired renal function was observed. Intervention causes a significantly lower serum creatinine, and cystatin-C concentration in the active treatment group compared with those on placebo (*p* = 0.0002 and *p* = 0.001 resp.). The evaluation with CKD-EPI based on both creatinine and cystatin-C showed a corresponding significant difference (*p* < 0.0001). All validations showed corresponding significant differences. In individuals with a deficiency of selenium and coenzyme Q_10,_ low selenium status is related to impaired renal function, and thus supplementation with selenium and coenzyme Q10 results in significantly improved renal function as seen from creatinine and cystatin-C and through the CKD-EPI algorithm. The explanation could be related to positive effects on inflammation and oxidative stress as a result of the supplementation.

## 1. Introduction

Renal function is of fundamental importance to the health of the body. In states with an impaired renal function, the impacts can be seen in several organ systems. The cardiovascular system is one where an impaired renal function will have important effects both in terms of prognosis, but also in the choice of treatment in most heart diseases. Due to the intimate relationship between the two organ systems, the concept of the cardiorenal syndrome has been developed [1]. As inflammation is one of the major drivers for most heart diseases [2,3,4], it is interesting to note that most renal diseases are also associated with inflammation [5,6,7]. Moreover, in early stages of renal dysfunction, when the glomerular function is still intact, systemic and local inflammation leads to downregulation of molecules that have renal protective effects [8].

Selenium is one of the essential trace elements needed to obtain normal cellular functions [9,10]. The cell needs selenium in many processes, for example in the defence against infections and selenium also functions in the energy transfer. Due to low selenium content of the soil in Europe, and many other parts of the world, there is a wide-spread low dietary selenium intake, which in some regions, results in a selenium deficiency. The estimated selenium intake in Europe is <50 μg/day [11]. In order to reach an optimal function of one of the most important selenoproteins, selenoprotein P, an intake of around 100 μg/day, or more is needed for an adult Caucasian [12]. A selenium deficiency has consequences resulting in increased risk of cardiovascular disease [13], among many other effects. Also, in conditions with increased oxidative stress and inflammation, the need for selenium is increased [14]. The kidneys, together with the thyroid gland, have the highest concentration of selenium in the body [15]. It has been shown that in chronic kidney disease, the concentration of selenium is lower compared with healthy individuals [16,17,18]. In a mouse model, selenium deficiency caused renal injury through increased oxidative stress, and impaired mitochondrial function [19].

Furthermore, it has been reported that there is a protective effect of selenium on ischaemic injuries on the kidney in a rat model [20]. In situations with toxic damage to the renal function, caused by severe nephrotoxic metals such as cadmium, lead, mercury and cisplatin [21], protective effects of selenium have been reported [22,23]. Liu et al. also reported attenuation of oxidative damage and inflammation caused by mycotoxin T-2 as an effect of selenium intervention in a rat model [24].

Coenzyme Q_10_ is also necessary for optimal cellular function. It is present in the mitochondrial respiratory chain, and it is also one of the most powerful intracellular lipid soluble antioxidants. As the endogenous production of coenzyme Q_10_ declines with increasing age, the resultant myocardial production of coenzyme Q_10_ at the age of 80 years, is only half of that obtained at the age of 20 years [25]. 

The cytosolic selenoenzyme thioreductase1 plays an important part in the reduction of ubiquinone to ubiquinol, which is the active form of coenzyme Q_10_. Therefore, there is an important interrelationship between selenium and coenzyme Q_10_ that explains the need for an optimal level of both substances in order to ensure a normal cellular function [26].

Positive effects of intervention with ubiquinol on the endothelial function in individuals with dyslipidaemia have also been reported [27]. In septic rats, improved organ and vessel function have been reported as a result of supplementation with coenzyme Q_10_ [28]. From scintigraphic evaluations of renal ischaemic injuries, protective effects have been reported by the intervention with coenzyme Q_10_ based mainly on the antioxidant effects of the substance [29]. Therefore, we hypothesise that the two substances, selenium and coenzyme Q_10_, might have a positive effect on the renal function in elderly individuals living in areas with a low selenium concentration in the soil and showing decreased endogenous synthesis of coenzyme Q10. 

There are several algorithms to estimate GFR in humans. We have chosen the Chronic Kidney Disease Epidemiology Collaboration (CKD-EPI) algorithm, one of the most widely used algorithms. We have applied both the algorithm using creatinine, and the algorithm using cystatin-C, and also the algorithm using both variables in our evaluation. Generally, in the elderly population, the creatinine-based algorithm provides acceptable sensitivity and specificity. However, in those with a low GFR, or on treatment with specific medication, the creatinine-based algorithm is not recommended, why we have added the cystatin-C based algorithm. As mentioned in the literature, the combined algorithm might give better precision, and we have also added this algorithm in our evaluations.

The aim of the present study was two-fold: First, to investigate whether selenium status is associated with renal function in an elderly Swedish community-living population, and secondly, in a sub-study to investigate a possible influence of supplementation over four years with selenium and coenzyme Q_10_ on the renal function. 

## 2. Materials and Methods 

### 2.1. Subjects

All persons (*n* = 1320) living in a specific municipality, in the age stratum 69–88 years, were invited in 1998 to participate in an epidemiological project. Out of these 876 decided to participate in the main epidemiological project. In 2003, 675 individuals were still alive and were invited to participate in an intervention project with selenium and coenzyme Q10 as a dietary supplement. As some individuals regarded the transportation distance to the Health Center for inclusion in this sub project too long, those who accepted participation were 589 individuals, 270 men and 319 women, aged 69–87 years. All persons attended a clinical examination and delivered blood samples. Among these individuals, we investigated whether plasma selenium was associated with serum creatinine as a measure of renal function. Out of the 586 individuals, 443 community-living persons in the age range of 70–88 years agreed to participate in the project, which involved supplementation with selenium and coenzyme Q_10_, or placebo and a follow-up programme for four years, which included blood samples every 6 months [30].The participants in the intervention study had a deficient pre-intervention serum selenium concentration, mean 67 μg/L (SD 16.8) (equivalent to an estimated daily intake of 35 μg/day), which is well below an adequate selenium concentration of ≥100 μg/L [31]. 

The subjects were given dietary supplementation of 200 mg/day of coenzyme Q_10_ capsules (Bio-Quinon 100 mg B.I.D, Pharma Nord, Vejle, Denmark) and 200 µg/day of organic selenium yeast tablets (SelenoPrecise 100 µg B.I.D, Pharma Nord, Vejle, Denmark) (*n* = 221), or a similar placebo (*n* = 222) over 48 months, after which the intervention was finished. The given supplementation was taken in addition to regular medication, if used. All study medications (active drug and placebo) not consumed were returned and counted. The participants were all examined by one of three experienced cardiologists. A clinical history was recorded at inclusion, and a clinical examination was performed at inclusion and after the study period, including blood pressure, assessment of New York Heart Association functional class (NYHA class) as well as an electrocardiogram (ECG) and Doppler-echocardiography. Echocardiographic examinations were performed with the participant in the left lateral position. The ejection fraction (EF) readings were categorised into four classes with interclass limits placed at 30%, 40% and 50% [32,33]. Normal systolic function was defined as EF ≥ 50%, while severely impaired systolic function was defined as EF *<* 30%. Only the systolic function was evaluated. The inclusion started in January 2003 and finished in February 2010. 

As the sub-analysis of the current study only included those who agreed to provide blood samples during the whole intervention period, and who also survived for the total intervention period, the final study population presented here consisted of 215 individuals of which 117 individuals received active supplementation with selenium and coenzyme Q10 and 98 individuals received a placebo (Table 1).

### 2.2. Ethical Approval 

The study was approved by the Regional Ethical Committee (Forskningsetikkommmitten, Hälsouniversitetet, SE-581 85 Linköping, Sweden; No. D03-176), and conforms to the ethical guidelines of the 1975 Declaration of Helsinki. (The Medical Product Agency declined to review the study protocol since the study was not considered a trial of a medication for a certain disease, but rather one of food supplement commodities that are commercially available). This study was registered at Clinicaltrials.gov, and has the identifier NCT01443780. Since it was not mandatory to register at the time the study began, the study has been registered retrospectively. Written, informed consent was obtained from all patients. 

### 2.3. Blood Sampling 

All blood samples were collected at inclusion in the study, and after 48 months. They were taken while the participants were resting in a supine position. Pre-chilled, EDTA vials for plasma were used. The vials were centrifuged at 3000× *g*, +4 °C, and were then frozen at −70 °C. No sample was thawed more than once.

### 2.4. Determination of Selenium

The serum selenium analyses were performed using ICP-MS methodology on an Agilent 700 platform at Kompetenzzentrum für komplementärmedizinische Diagnostik, Zweigniederlassung der synlab MVZ Leinfelden GmbH (Leinfelden-Echterdingen, Germany). The accuracy of the measurements was checked by analysing two external reference materials with certified values of 63 μg/L and 103 μg/L (control programme offered by the Society for Advancement of Quality Assurance in Medical Laboratories, INSTAND e.V., Düsseldorf, Germany), showing values within 90–110% of certified concentrations. A round-robin test with INSTAND e.V. was always passed adequately. The precision of the method, checked by repetitive analyses of the same sera, showed an average coefficient of variation of 5.7%. 

### 2.5. Determination of Creatinine and Cystatin-C

Creatinine and cystatin-C were analysed on a Cobas c701 chemistry analyser (Roche Diagnostics, Rotkreutz, Switzerland) with reagents from the same manufacturer. The creatinine method used was enzymatic and isotope dilution mass spectrometry was calibrated. Cystatin-C was analysed with a particle enhanced turbidimetric assay.

### 2.6. Determination of Renal Function

It is well-known that the serum concentration of creatinine is influenced by the total muscle mass of the individual, which in this case could change in the elderly over the five years of intervention. It is also influenced by gender and age. Therefore, cystatin-C could be a better measure of kidney function. We have thus evaluated cystatin-C in the same population.

To adjust for gender and age and race, appropriate algorithms have been proposed. The CKD-EPI is one of the most widely used [34]. Applying the obtained data to the algorithm we used both the CKD-EPI based on creatinine, and on cystatin-C. 

However, in an elderly population, e-GFR based on cystatin-C is generally seen as a better alternative as compared to e-GFR based on creatinine alone [35,36]. Therefore, we performed evaluations based on cystatin-C concentration when evaluating the e-GFR as well.

For an adult European population, the use of the combined CKD-EPI based on both creatinine and cystatin-C has been recommended [37]. In all evaluations described above, the group on active treatment demonstrated a better renal function after five years of follow-up, as compared to those on placebo.

eGFR_Creatinine_ was estimated in mL/min/1.73 m^2^ using the Chronic Kidney Disease Epidemiology Collaboration (CKD-EPI) creatinine equation from 2009 [38].
eGFR_Cystatin C_ in mL/min/1.73 m^2^ was calculated from cystatin-C using the CKD-EPI cystatin-C equation for estimating GFR with standardised serum cystatin-C values [39].
eGFR_Combined_ in mL/min/1.73 m^2^ was calculated using the CKD-EPI combined creatinine/cystatin-C equation [40].

### 2.7. Statistical Methods 

In the evaluation of a possible association between the concentration of selenium in serum and the serum level of creatinine, Pearson product-moment correlation analysis was performed, in order to evaluate a possible influence of other covariates a multiple regression applying creatinine as the dependent variable was performed.

In the intervention sub-study, descriptive data were presented as percentages or mean ± standard deviation (SD). A Student’s unpaired two-sided T-test was used for continuous variables and the chi-square test was used for analysis of one discrete variable. Repeated measures of variance were used in order to obtain better information on the individual changes in the concentration of the biomarker analysed, compared to group mean values.

In the analysis of covariance (ANCOVA) evaluation, both transformed and non-transformed data were applied, with no significant difference in the results. 

In the ANCOVA evaluation, the creatinine concentration, cystatin-C concentration, CKD-EPI based on both creatinine and cystatin-C, CKD-EPI based on creatinine, and finally CKD-EPI based on cystatin-C, after 48 months were used as dependent variables in the respective models. In each model, adjustments were made for age, smoking, hypertension, diabetes, ischaemic heart disease (IHD), New York Heart Association (NYHA) class III, Hb < 120 g/L and selenium concentration in serum at inclusion. 

*p*-values < 0.05 were considered significant, based on a two-sided evaluation. All data were analysed using standard software (Statistica v. 13.2, Dell Inc., Tulsa, OK, USA).

## 3. Results

### 3.1. Association Between Serum Selenium and Renal Function

As a first step we wanted to establish if there was any relation between renal function and selenium before any intervention. In an unadjusted regression analysis in the population of 589 individuals we observed a moderate negative correlation between selenium and renal function as measured through creatinine (*r* = −0.52; F: 213.6; *p* < 0.000). The significant association was verified in a Pearson product-moment correlation analysis (*r* = 0.52). To investigate further this apparent association between the serum concentration of selenium and creatinine we adjusted for covariates well-known to potentially influence the renal function, employing a multiple regression using creatinine as the dependent variable (Table 2). 

Also, in this latter analysis, we found a strong association between selenium concentration and creatinine.

### 3.2. Intervention with Selenium and CoQ_10_, and Impact on Renal Function 

The final study population presented here consisted of 215 individuals, of which 117 received active supplementation with selenium and coenzyme Q_10_ and 98 received a placebo (Table 1). From Table 1 it can be seen that 37 out of 215 (17.2%) had diabetes, 152 out of 215 (70.7%) had hypertension, 38 (17.7%) had an IHD, and 11 (5.1%) had an impaired systolic heart function, here defined as an EF < 40%. It could therefore be seen that the investigated population represented an elderly community-living Western population. There were no significant differences between those randomised to active substance, when compared with those randomised to placebo; thus the two populations were balanced. Regarding the mean pre-intervention concentration of both selenium and coenzyme Q_10_, they were low, but there were no significant differences between the active treatment group and those on placebo.

The mean follow-up period of this sub-population was 5.1 years.

### 3.3. Evaluating the Effect of Intervention on Creatinine

At baseline there was no significant difference in the serum concentrations of creatinine between the active treatment group and those receiving placebo (active: 92.3 µmol/L vs. placebo: 90.8 µmol/L; *p* = 0.72). However, after 48 months of intervention, a significantly lower concentration of creatinine could be noted in the active treatment group, whereas there was no difference in the placebo group (active: 76.8 µmol/L vs. placebo: 90.5 µmol/L; T = 3.83; *p* = 0.0002).

As the sample size of the study population was limited, we performed a two-step validation.

**Figure 1:**
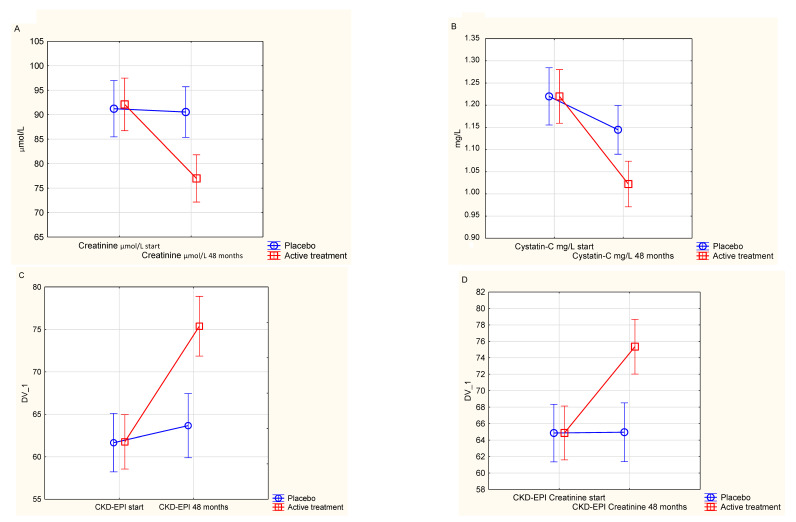
(**A**) Serum concentration of creatinine at the start of the project and after 48 months in the selenium and coenzyme Q_10_ treatment group compared to the placebo group in the study population. Evaluation performed by use of repeated measures of variance methodology. Current effect: F(1, 204) = 14.2; *p* = 0.0002. Vertical bars denote 0.95 confidence intervals. Blue curve: Placebo; Red curve: Active treatment group. Bars indicate ±95% CI; (**B**) Serum concentration of cystatin-C at the start of the project and after 48 months in the selenium and coenzyme Q_10_ treatment group compared to the placebo group in the study population. Evaluation performed by use of repeated measures of variance methodology. Current effect: F(1, 203) = 9.08; *p* = 0.003. Vertical bars denote 0.95 confidence intervals. Blue curve: Placebo; Red curve: Active treatment group. Bars indicate ±95% CI. (**C**) Estimated glomerular filtration rate based on the CKD-EPI algorithm using both creatinine and cystatin-C comparing values in the selenium and coenzyme Q_10_ treatment group compared to the placebo group in the study population, at inclusion and after 48 months of intervention. Evaluation performed by use of repeated measures of variance methodology. Current effect: F(1, 202) = 23.70; *p* < 0.0001. Vertical bars denote 0.95 confidence intervals. Blue curve: Placebo; Red curve: Active treatment group. Bars indicate ±95% CI. (**D**) Estimated glomerular filtration rate based on the CKD-EPI algorithm using creatinine, comparing values in the selenium and coenzyme Q_10_ treatment group compared to the placebo group in the study population, at inclusion and after 48 months of intervention. Evaluation performed by use of repeated measures of variance methodology. Current effect: F(1, 204) = 16.68; *p* < 0.0001. Vertical bars denote 0.95 confidence intervals. Blue curve: Placebo; Red curve: Active treatment group. Bars indicate ±95% CI. CKD-EPI: Chronic Kidney Disease Epidemiology Collaboration eGFR algorithm. Vertical bars denote 0.95 confidence intervals.

First, as a validation, a repeated measures of variance analysis was performed (Figure 1A). A clearly significant difference between the two groups could be noted (F(1, 204) = 14.2; *p* = 0.0002).

As a second validation of the obtained results, an ANCOVA analysis, was performed (Table 3).

From this we could see that after adjustment for several covariates, the supplementation with selenium and coenzyme Q_10_ still had a significant effect on the concentration of creatinine.

### 3.4. Evaluating the Effect of Intervention on Cystatin-C

As cystatin-C is increasingly used as a reliable biomarker for evaluating the renal function, we chose to include this analysis in the study. At the start of the intervention, there was no significant difference in the serum concentration of cystatin-C between the two groups (active: 1.23 mg/L vs. placebo: 1.22 mg/L; *p* = 0.97). However, after the intervention, a significant reduction could be noted in the active treatment group. Whereas, there was no significant change in the placebo group (active: 1.02 mg/L vs. placebo: 1.14 mg/L; T = 3.30; *p* = 0.001).

As a validation of the obtained differences, a repeated measures of variance analysis was performed. A significant difference could be demonstrated, with a lower concentration in the active treatment group compared to the placebo group (F(1, 203 = 9.08; *p* = 0.003)) (Figure 1B).

In the second validation, the ANCOVA analysis adjustments were made for some covariates well-known to influence renal function (Table 4). 

Here it was shown that active treatment did significantly influence the level of cystatin-C (*p* = 0.0003), even in a multivariate model.

### 3.5. Evaluating the Effect of Intervention on CKD-EPI Based on Both Creatinine and Cystatin-C

The CKD-EPI equation is one of the most commonly used formulas to estimate the renal glomerular function. We have applied the recorded basal data of the study population to obtain the estimated glomerular filtration rate by using this formula.

At start of the intervention, there were no significant differences between those randomised to active treatment, and those randomised to placebo (active: 61.4 mL/min/1.73 m^2^ versus placebo: 61.8 mL/min/1.73 m^2^; *p* = 0.88). However, after 48 months of intervention, a significant increase in e-GFR could be seen in the active treatment group (48 months: 75.4 mL/min/1.73 m^2^ versus inclusion: 61.4 mL/min/1.73 m^2^; T = 4.50; *p* < 0.0001). No significant changes were noted in the placebo group (48 months: 63.7 mL/min/1.73 m^2^ vs. inclusion: 61.8 mL/min/1.73 m^2^; *p* = 0.45).

To validate the obtained results, repeated measures of variance methodology was applied. From this a significant difference between those on active treatment and those on placebo could be demonstrated (F(1, 202) = 23.7; *p* < 0.0001) (Figure 1C).

As a second validation, ANCOVA evaluation was performed, including CKD-EPI based on both creatine and cystatin-C (Table 5). 

From that it could be noted that supplementation with selenium and coenzyme Q_10_ had a significant effect on the change of e-GFR as observed through the CKD-EPI calculation (F = 25.56; *p* < 0.0001).

### 3.6. Evaluating the Effect of Intervention on CKD-EPI Based on Creatinine

As the CDK-EPI formula can also be evaluated based on serum creatinine concentration alone, without the cystatin-C levels, we have calculated these data as well.

At inclusion, there were no significant differences between the e-GFR given as CKD-EPI, based on the creatinine concentration (active treatment: 64.5 mL/min/1.73 m^2^ vs. placebo: 65.1 mL/min/1.73 m^2^; *p* = 0.82). However, after 48 months of intervention, a significant increase of the e-GFR could be seen in the active treatment group (48 months: 75.4 mL/min/1.73 m^2^ vs. incl.: 64.5 mL/min/1.73 m^2^; T = 4.92; *P* ≤ 0.0001). There was no significant change in the placebo group 64.9 mL/min/1.73 m^2^ vs. 65.1 mL/min/1.73 m^2^; *p* = 0.96).

By applying repeated measures of variance methodology, significant differences between the active treatment group and the placebo group could be seen (F(1, 204) = 16.68; *p* < 0.0001) (Figure 1D).

Performing the second validation step, that is the ANCOVA analysis, it was found that active treatment significantly influenced the CKD-EPI value, based on creatinine (F = 20.63; *p* < 0.0001) (Table 6).

### 3.7. Evaluating the Effect of Intervention on CKD-EPI Based on Cystatin-C

As the CKD-EPI value could also be based on the cystatin-C concentration alone, we also calculated the latter.

At the start of the project, there was no significant difference between the active treatment group, and the placebo group (active: 58.3 mL/min/1.73 m^2^ vs. placebo: 59.2 mL/min/1.73 m^2^; *p* = 0.71). After 48 months, a significant increase in the CKD-EPI value could be noted (48 months: 72.6 mL/min/1.73 m^2^ vs. incl.: 58.3 mL/min/1.73 m^2^: T = 5.76; *p* < 0.0001). In the placebo group we observed no significant change (48 months: 63.7 mL/min/1.73 m^2^ vs. incl.:59.2 mL/min/1.73 m^2^; *p* = 0.11).

**Figure 2:**
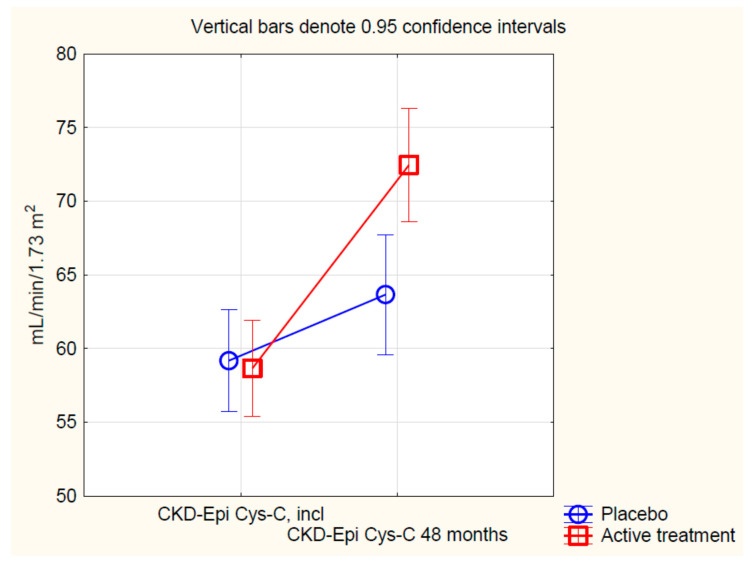
Estimated glomerular filtration rate based on the CKD-EPI algorithm using cystatin-C, comparing values in the selenium and coenzyme Q_10_ treatment group compared to the placebo group in the study population, at inclusion and after 48 months of intervention. Evaluation performed by use of repeated measures of variance methodology. Current effect: F(1, 204) = 14.3; *p* = 0.0002. Vertical bars denote 0.95 confidence intervals. Blue curve: Placebo; Red curve: Active treatment group. Bars indicate ±95% CI. CKD-EPI: Chronic Kidney Disease Epidemiology Collaboration eGFR algorithm

Applying repeated measures of variance methodology, a highly significant difference could be seen (F(1, 204) = 14.3; *p* = 0.0002) (Figure 2), where those on active treatment exhibited higher e-GFR as a result of the treatment.

By applying ANCOVA evaluation as a measure to adjust for several covariates that could influence renal function, it could be noted that active treatment influenced the e-GFR even after adjustments for several covariates (F = 13.9; *p* = 0.0002) (Table 7).

### 3.8. Impact of Pre-Intervention Selenium Concentration on the Effect of Supplementation on the Renal Function

In an effort to evaluate whether the effect of the supplementation of selenium and coenzyme Q10 on renal function was dependent on the level of selenium before any supplementation, we compared the improvement in renal function of those with a selenium level in the first quartile with that of those in the fourth quartile. As a measure of renal function, we chose CKD-EPI creatinine. We observed a significantly increased renal function in both groups by applying repeated measures of variance methodology. F(1, 63) = 5.21; *p* = 0.026 and F(1, 50) = 4.46; *p* = 0.04, in the first and fourth quartiles, respectively. We then used the difference in creatinine as a measure of renal improvement, and found that the mean difference in creatinine in the group with the10% lowest pre-intervention selenium concentration (<42.8µg/L) was 20.55 µmol/L as compared to 9.3 µmol/L among those with the highest 10% of selenium concentration (>83.8 µg/L). Both groups consisted of 11 individuals and, not surprisingly, the difference between the groups was not significant (*p* = 0.30). However, because of the small number, we could not rule out that the impact of the intervention might nevertheless be dependent on pre-inclusion selenium status. 

### 3.9. Analysing the Effect of Supplementation Based on Pre-Intervention eGFR

In order to analyse whether the pre-intervention eGFR influenced the effect of the supplementation with selenium and coenzyme Q10, repeated measure of variance analyses of the effect of the supplementation were performed based on CKD-Epi creatinine algorithm (Supplemental Figure S1). From this it could be demonstrated that with no major difference in effect could be seen in the tertiles of the eGFR. 

## 4. Discussion

The two important findings in this elderly community-living population low in selenium and coenzyme Q_10_ were the finding of an association between low selenium status and impaired renal function and that dietary supplementation with selenium and coenzyme Q_10_ over four years significantly improved the kidney function. There was no change in the kidney function of the placebo group. The results of the intervention provide evidence of a causal relationship between selenium status and renal function among elderly low in selenium and CoQ_10_.

As the intervention has made use of a relatively small sample size, a two-step validation was performed in all of the evaluations. Also, in the validation steps a significantly better renal function could be seen in the active treatment group, as compared to those on placebo.

The mechanism behind the demonstrated improvement in renal function in the supplemented group is believed to be due partly to the reduced inflammation, as well as reduced oxidative stress, which we have observed among this elderly group in previous studies [41,42,43]. 

The main study reported increased cardiac systolic function, reduced level of the cardiac peptide NT-proBNP, and reduced cardiovascular mortality [30]. Therefore, some of the present observations on improved renal function could be a result of the improved systolic cardiac function, resulting in a better cardiac output, which improves the renal function.

Our group has also reported signs of less endothelial dysfunction otherwise accompanying ageing, due to the intervention with selenium and coenzyme Q_10_, as seen from evaluation of the von Willebrand factor, and the plasminogen activator inhibitor-1 [44] and the increase in IGF-1 [45]. Therefore, it seems that in a population with deficiency, supplementation with selenium and coenzyme Q_10_ reduces the inflammatory activity and oxidative stress, and improves endothelial function. These mechanisms may, thus, be important for the observed improvement in kidney function.

Renal function is of fundamental importance in the health of the body, and also influences many treatment decisions, for example in cardiovascular diseases. The literature provides evidence of an intimate relationship between inflammation and mRNA expression of selenoproteins which influence the synthesis of several inflammatory factors, for example IL-6, IL-8, IL-12 cyclooxygenase-2, IL-10, and TGF-β [46,47], also resulting in effects on the renal function. Our results accord well with reports in the literature demonstrating a relationship between selenium concentration and progression of kidney disease [48]. 

Recently, Li et al. reported from a study on pigs that selenium deficiency resulted in inflammatory injuries and renal tubular atrophies, leading to impaired kidney function, and as a result of the selenium deficiency, downregulation of nine different selenoproteins [47]. The result described concurs with our results.

Regarding coenzyme Q10, interesting data have been reported in the literature indicating that coenzyme Q10 prevents the adverse effects of oxidative stress [49], and that oxidative stress has adverse effects on renal function [50]. 

An interesting aspect of the relation between selenium concentration and renal health is the importance of endothelial dysfunction in the progression of chronic kidney disease [51,52]. There is an important and well-known association between kidney disease and cardiovascular risk [53,54,55], which is in accordance with the present observations on renal protection, as selenium deficiency results in increased cardiovascular risk [56]. Another interesting aspect might also be the findings that our group previously reported from a metabolic profiling evaluation of the effects of the supplementation [57]. Here, a small, but significant, change in the metabolites reflecting the renal function could be seen, indicating a positive effect of the supplementation also on the renal function.

In an effort to determine whether the basal selenium concentration influenced the effect on the renal function due to the supplementation, those with the lowest selenium concentration were compared with those with the highest selenium concentration. We, then, observed that the most selenium-deficient group achieved the greatest improvement in renal function. However, those in this study population who had the highest selenium concentration also gained in renal function as a result of the supplement. It should be noted, however, that those who had a selenium concentration in the fourth quartile, were still low on selenium (mean level 74.9 µmol/L), which might explain the positive effect of the supplementation also in this group.

Taken together, the findings demonstrate that the combined supplementation with selenium and coenzyme Q_10_ in a region where there is a suboptimal intake of selenium could improve renal function. However, the presented study is small, and should be regarded as hypothesis-generating, and therefore more research is needed.

### Limitations

As the investigated study population consists of a relatively narrow age stratum, it is not possible to extrapolate the results to other age groups without uncertainty.

The study sample that is analysed in this report was of relatively small size, which increases the uncertainty of the obtained results. However, we argue that the results are likely to be correct as they were validated by the two-step validation analysis. Nevertheless, based on the small sample size we consider the results as hypothesis-generating.

Also, the evaluated population consisted of Caucasians who were low in selenium. Therefore, it is not necessarily true that the obtained results could be applied to another population.

## 5. Conclusions

Renal function is central maintaining health. Selenium and coenzyme Q10 are necessary to maintain normal cellular functions. As there is a documented deficiency of both substances in the elderly in many areas in the world, the effect of supplementation with selenium and coenzyme Q10 on renal function was evaluated.

Significantly better renal function in the active treatment group, as seen by evaluation of both creatinine and cystatin-C, could be reported. An estimation of glomerular filtration rated by use of CKD-EPI verified the positive effects. All evaluations were validated with persisting differences. 

The obtained positive results could possibly be explained by effects on inflammation and oxidative stress by the intervention, but as the sample size was small, more research is needed.

## Figures and Tables

**Table 1:** Baseline characteristics of the study population receiving active treatment or placebo during an intervention time of four years.

	Active Treatment Group *n* = 117	Placebo Group *n* = 98	*p*-Value
Gender, Males/Females	58/59	44/54	
Age years, mean (SD)	76.2 (3.1)	76.2 (3.1)	0.96
**History**			
Smoking, *n* (%)	8 (6.8)	9 (9.2)	0.53
Hypertension, *n* (%)	81 (69.2)	71 (72.4)	0.61
IHD, *n* (%)	22 (18.8)	16 (16.3)	0.64
Diabetes, *n* (%)	20 (17.1)	17 (17.3)	0.96
NYHA class I, *n* (%)	72 (61.5)	57 (58.2)	0.61
NYHA class II, *n* (%)	28 (23.9)	30 (30.6)	0.27
NYHA class III, *n* (%)	17 (14.5)	10 (10.2)	0.34
Unclassified, *n* (%)	0	1	-
**Medications**			
Beta blockers, *n* (%)	42 (35.9)	33 (33.7)	0.73
ACEI/ARB, *n* (%)	19 (16.2)	21 (21.4)	0.33
Digitalis, *n* (%)	5 (4.3)	1 (1.0)	-
Diuretics, *n* (%)	37 (31.6)	33 (33.7)	0.75
Statins, *n* (%)	27 (23.1)	18 (18.4)	0.40
**Examinations**			
EF < 40%, *n* (%)	7 (6.0)	4 (4.1)	-
s-selenium pre-intervention µg/L, mean (SD)	65.0 (17.5)	65.2 (17.1)	0.91
s-coenzyme Q10 pre-intervention mg/L, mean (SD)	0.80 (0.30)	0.83 (0.34)	0.65

ACEI: ACE- inhibitors; ARB: Angiotension receptor blockers; EF: Ejection fraction; IHD: Ischemic heart disease; NYHA: New York Heart Association functional class; SD: Standard Deviation. Values are means ± SDs or frequency (percent). Student’s unpaired two-sided T-test was used for continuous variables and the chi-square test was used for analysis of one discrete variable.

**Table 2:** Multiple regressions analysis using creatinine as dependent variable, adjusting for covariates well-known to potentially influence the creatinine level.

Variable	Β-Coefficient	Standard Error	T-Value	*p*-Value
Age	0.25	0.03	7.16	<0.0001
Male gender	0.25	0.03	7.30	<0.0001
Smoking	0.05	0.03	1.50	0.13
Diabetes	0.02	0.03	0.73	0.46
Hypertension	−0.05	0.03	−1.49	0.14
Hb < 120 g/L	0.03	0.003	0.97	0.33
NYHA III	−0.002	0.003	−0.05	0.96
IHD	−0.04	0.04	−1.00	0.32
ACEI	0.05	0.03	1.59	0.11
Beta blockers	0.04	0.03	1.03	0.30
Selenium, incl.	−0.47	0.03	14.2	<0.0001

ACEI: Angiotensin converting enzyme inhibitor; IHD: Ischemic heart disease; NYHA III: New York Heart Association functional class III.

**Table 3:** Analysis of covariance using s-creatinine after 48 months as dependent variable.

Effects	Degrees of Freedom	F	*p*
Intercept	1	0.01	0.91
Age	1	0.61	0.43
Smoker	1	0.03	0.86
Hypertension	1	2.07	0.15
Diabetes	1	0.13	0.71
IHD	1	1.66	0.20
NYHA III	1	0.98	0.32
Hb < 120 g/L	1	1.01	0.32
s-selenium µg/L, incl	1	1.40	0.24
s-creatinine, micromol/L, incl	1	61.28	<0.0001
Active treatment	1	18.11	<0.0001

IHD: Ischemic heart disease; NYHA: New York Heart Association functional class III.

**Table 4:** Analysis of covariance using s-cystatin-C after 48 months as dependent variable.

Effects	Degrees of Freedom	F	*p*
Intercept	1	0.01	0.90
Age	1	1.12	0.29
Smoker	1	0.05	0.29
Hypertension	1	3.70	0.06
Diabetes	1	0.19	0.66
IHD	1	0.08	0.78
NYHA III	1	1.72	0.19
Hb < 120 g/L	1	4.33	0.04
s-selenium µg/L, incl	1	0.07	0.79
s-cystatin-C, mg/L, incl	1	66.65	<0.0001
Active treatment	1	12.92	0.0004

IHD: Ischemic heart disease; NYHA: New York Heart Association functional class III.

**Table 5:** Analysis of covariance using CKD-EPI based on both creatinine and cystatin-C, after 48 months as dependent variable.

Effects	Degrees of Freedom	F	*p*
Intercept	1	9.64	0.002
Age	1	4.17	0.04
Smoker	1	0.05	0.83
Hypertension	1	6.97	0.009
Diabetes	1	0.01	0.92
IHD	1	0.95	0.33
NYHA III	1	0.98	0.32
Hb < 120 g/L	1	1.52	0.22
s-selenium µg/L, incl	1	0.001	0.97
CKD-EPI, mL/min/1.73 m^2^, incl	1	63.88	<0.0001
Active treatment	1	26.06	<0.0001

IHD: Ischemic heart disease; NYHA: New York Heart Association functional class III; CKD-EPI: Chronic Kidney Disease Epidemiology Collaboration eGFR algorithm. In this evaluation the CKD-EPI algorithm was based on both creatinine, and cystatin-C concentration in serum.

**Table 6:** Analysis of covariance using CKD-EPI based on creatinine after 48 months as dependent variable.

Effects	Degrees of Freedom	F	*p*
Intercept	1	12.53	0.0005
Age	1	4.73	0.03
Smoker	1	0.02	0.89
Hypertension	1	4.45	0.04
Diabetes	1	0.54	0.46
IHD	1	2.61	0.11
NYHA III	1	0.65	0.42
Hb < 120 g/L	1	0.25	0.62
s-selenium µg/L, incl	1	1.40	0.24
CKD-EPI, mL/min/1.73 m^2^, incl	1	41.92	<0.0001
Active treatment	1	20.63	<0.0001

CKD-EPI: Chronic Kidney Disease Epidemiology Collaboration eGFR algorithm; IHD: Ischemic heart disease; NYHA: New York Heart Association functional class III.

**Table 7:** Analysis of covariance using CKD-EPI based on cystatin-C after 48 months as dependent variable.

Effects	Degrees of Freedom	F	*p*
Intercept	1	6.69	0.01
Age	1	2.99	0.09
Smoker	1	0.20	0.65
Hypertension	1	5.31	0.02
Diabetes	1	0.27	0.61
IHD	1	0.09	0.76
NYHA III	1	1.05	0.31
Hb < 120 g/L	1	4.44	0.04
s-selenium µg/L, incl	1	0.31	0.58
CKD-EPI, mL/min/1.73 m^2^, incl	1	76.43	<0.0001
Active treatment	1	12.88	0.0004

CKD-EPI: Chronic Kidney Disease Epidemiology Collaboration eGFR algorithm; IHD: Ischemic heart disease; NYHA: New York Heart Association functional class III.

## Supplementary Materials

The following are available online at https://www.mdpi.com/2072-6643/12/12/3780/s1, Figure S1: Figure S1. (A) Estimated pre-intervention glomerular filtration rate based on the CKD-EPI algorithm using creatinine in tertile 1 of the study population, comparing values in the selenium and coenzyme Q_10_ treatment group with the placebo group, at inclusion and after 48 months of intervention. Evaluation performed by use of repeated measures of variance methodology. Current effect: F(1, 65) = 8.5882, *p* = 0.00466. Vertical bars denote 0.95 confidence intervals. Blue curve: Placebo; Red curve: Active treatment group. Bars indicate ±95% CI; (B) Estimated pre-intervention glomerular filtration rate based on the CKD-EPI algorithm using creatinine in tertile 2 of the study population, comparing values in the selenium and coenzyme Q_10_ treatment group with the placebo group, at inclusion and after 48 months of intervention. Evaluation performed by use of repeated measures of variance methodology. Current effect: F(1, 69) = 8.1391, *p* = 0.00571. Vertical bars denote 0.95 confidence intervals. Blue curve: Placebo; Red curve: Active treatment group. Bars indicate ±95% CI; (C) Estimated pre-intervention glomerular filtration rate based on the CKD-EPI algorithm using creatinine in tertile 3 of the study population, comparing values in the selenium and coenzyme Q_10_ treatment group with the placebo group, at inclusion and after 48 months of intervention. Evaluation performed by use of repeated measures of variance methodology. Current effect: Current effect: F(1, 64) = 10.087, *p* = 0.00230. Vertical bars denote 0.95 confidence intervals. Blue curve: Placebo; Red curve: Active treatment group. Bars indicate ±95% CI.

## Abbreviations

ACEI: ACE inhibitors; ANCOVA: Analysis of covariance; ARB; Angiotension receptor blockers; CKD-EPI: Chronic Kidney Disease Epidemiology Collaboration algorithm; EF: Ejection fraction; ECG: Electrocardiogram; GFR: Glomerular filtration rate; IHD: Ischaemic heart disease; NT-proBNP: N-terminal fragment of B-type natriuretic peptide; NYHA class: New York Heart Association functional class; SD: Standard deviation.
