# Supplementary material for: Selenium and Coenzyme Q10 Supplementation Improves Renal Function in Elderly Deficient in Selenium: Observational Results and Results from a Subgroup Analysis of a Prospective Randomised Double-Blind Placebo-Controlled Trial
Source: Nutrients. 2020 Dec 9;12(12):3780. doi: 10.3390/nu12123780 (PMC7764721; doi:10.3390/nu12123780)
Supplement: Supplementary file 1 [file nutrients-12-03780-s001.pdf]

A

Tertile 1

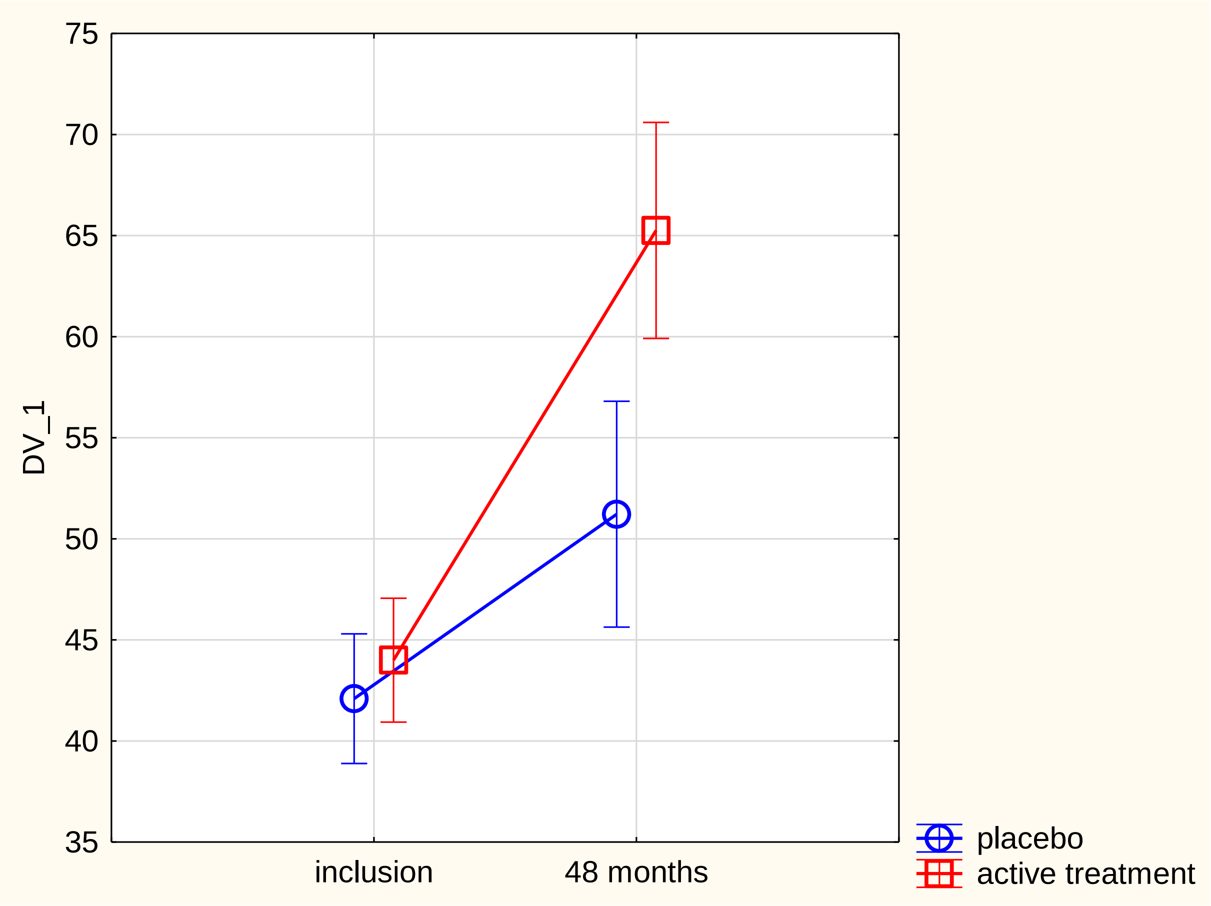

75
70
65
60
55
50
45
40
35
DV_1
inclusion
48 months
placebo
active treatment

B

Tertile 2

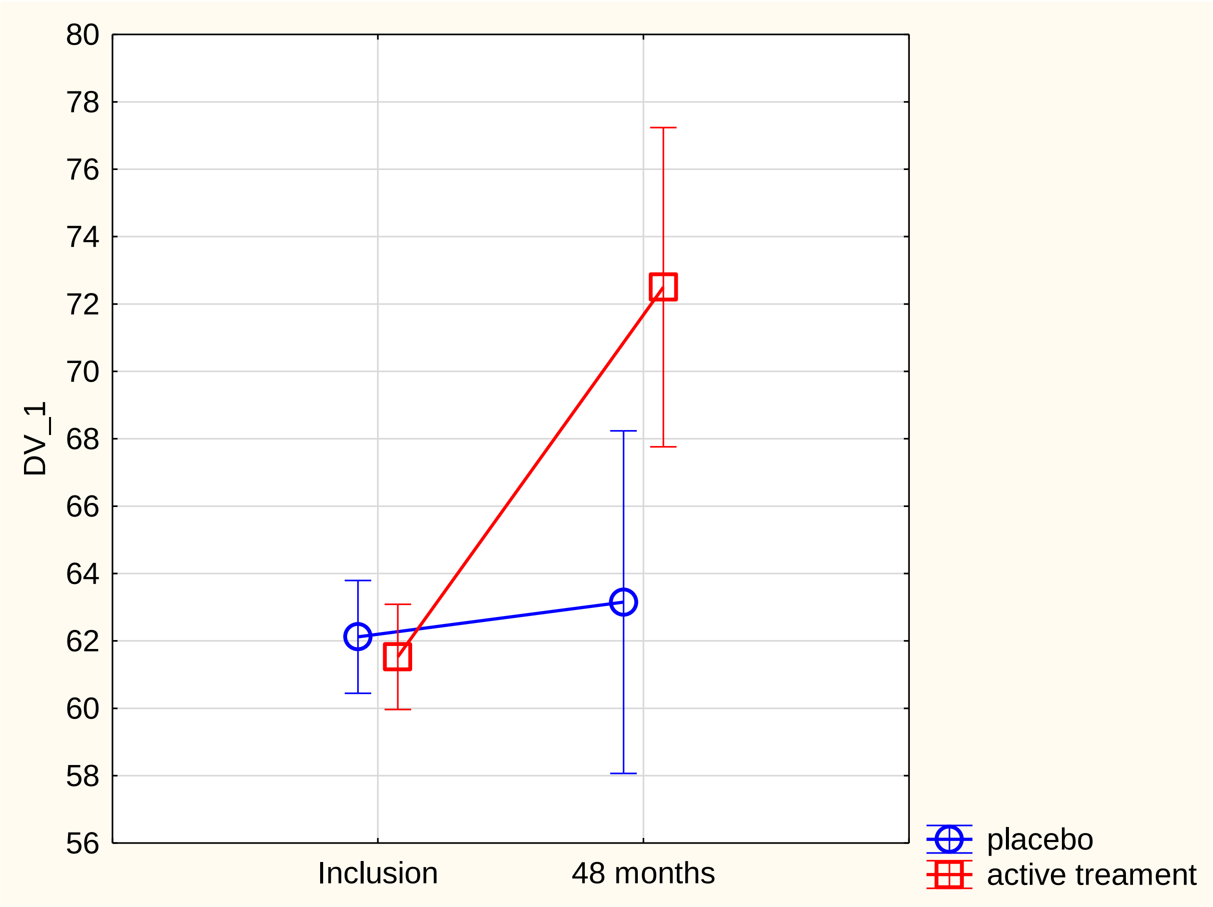
80
78
76
74
72
70
68
66
64
62
60
58
56
DV_1
Inclusion
48 months
placebo
active treament

C

Tertile 3

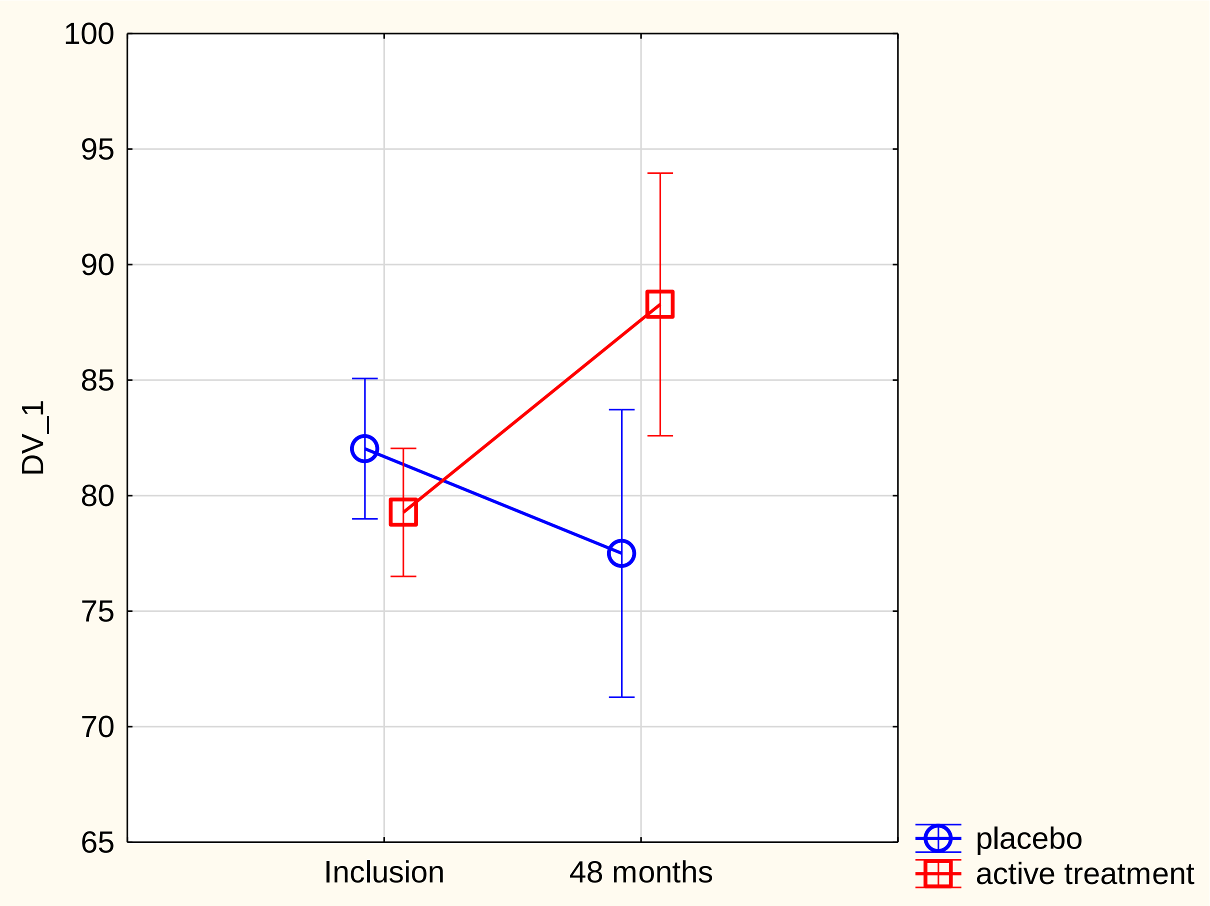

Figure S1. (A) Estimated pre-intervention glomerular filtration rate based on the CKD-EPI algorithm using creatinine in tertile 1 of the study population, comparing values in the selenium and coenzyme Q_{10} treatment group with the placebo group, at inclusion and after 48 months of intervention. Evaluation performed by use of repeated measures of variance methodology. Current effect: $F(1, 65) = 8.5882$, $p = 0.00466$. Vertical bars denote 0.95 confidence intervals. Blue curve: Placebo; Red curve: Active treatment group. Bars indicate ±95% CI; (B) Estimated pre-intervention glomerular filtration rate based on the CKD-EPI algorithm using creatinine in tertile 2 of the study population, comparing values in the selenium and coenzyme Q_{10} treatment group with the placebo group, at inclusion and after 48 months of intervention. Evaluation performed by use of repeated measures of variance methodology. Current effect: $F(1, 69) = 8.1391$, $p = 0.00571$. Vertical bars denote 0.95 confidence intervals. Blue curve: Placebo; Red curve: Active treatment group. Bars indicate ±95% CI; (C) Estimated pre-intervention glomerular filtration rate based on the CKD-EPI algorithm using creatinine in tertile 3 of the study population, comparing values in the selenium and coenzyme Q_{10} treatment group with the placebo group, at inclusion and after 48 months of intervention. Evaluation performed by use of repeated measures of variance methodology. Current effect: Current effect: $F(1, 64) = 10.087$, $p = 0.00230$. Vertical bars denote 0.95 confidence intervals. Blue curve: Placebo; Red curve: Active treatment group. Bars indicate ±95% CI.
